# A Modified Anatomical All‐Inside Double‐Bundle Anterior Cruciate Ligament Reconstruction Technique

**DOI:** 10.1002/atn2.70172

**Published:** 2026-06-10

**Authors:** Hanting Yi, Hongyu Li, Yaoqiang Chen, Wen Yang, Wentao Zhang, Mengjun Ma, Yundan Jiang

**Affiliations:** ^1^ Department of Orthopedics The Eighth Affiliated Hospital of Sun Yat‐sen University Shenzhen China; ^2^ Department of Radiology The Eighth Affiliated Hospital of Sun Yat‐sen University Shenzhen China

## Abstract

Arthroscopic anterior cruciate ligament (ACL) reconstruction (ACLR) is the standard surgical treatment for ACL injuries and is most commonly performed using anatomical single‐bundle (SB) or double‐bundle (DB) techniques. With the development of the all‐inside technique, the all‐inside SB technique using socket‐based tunnels and cortical suspensory fixation has gained popularity because it helps preserve bone stock, reduces postoperative pain, and provides reliable knee stability. Compared with the SB technique, the DB technique achieves comparable clinical outcomes, more closely replicates native ACL anatomy, and may provide superior anterior and rotational stability, lower revision rates, and higher return‐to‐sport rates in athletes and other high‐demand patients. However, the conventional DB technique requires substantial bone removal, is prone to tunnel widening or coalescence, and uses graft tissue less efficiently. To address these limitations, we describe a modified anatomical all‐inside DB technique that combines the advantages of the all‐inside technique and DB technique. This technique employs 4 bone sockets with suspensory fixation and bundle‐specific tensioning to preserve bone stock, optimize graft utilization, and facilitate anatomic DB reconstruction.

## Graphical abstract

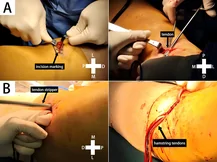

**VIDEO 1:** This video shows a modified anatomical all‐inside double‐bundle anterior cruciate ligament reconstruction using hamstring tendon autografts with socket‐based tunnels and cortical suspensory fixation. The left knee is shown. The procedure is performed with the patient supine and the operative knee prepared and draped in the standard fashion. Arthroscopy is carried out with a 30° arthroscope through 3 portals: an anterolateral viewing portal, an anteromedial portal, and an accessory anteromedial portal. After the surgical indications are reviewed, hamstring tendons are harvested through a 3 cm oblique incision made approximately 2 cm medial to the tibial tubercle and 4 cm distal to the joint line. Two grafts are prepared for the anteromedial bundle (AMB) and posterolateral bundle (PLB), each attached to adjustable‐loop titanium cortical buttons for femoral‐ and tibial‐side suspensory fixation. Femoral socket preparation is performed first with the knee flexed to approximately 90° under direct arthroscopic visualization. The PLB femoral socket is referenced to the intersection of the intercondylar ridge and the coronal plane through the anterior margin of the posterior horn of the lateral meniscus. The AMB femoral socket is referenced to the intersection of the posterior condylar cartilage margin and the intercondylar ridge. Pilot holes are created with a microfracture awl, and inside‐out drilling is performed in deep flexion using an anterogradely advanced 4.0 mm guide pin as a guide. Socket lengths are measured, and sequential inside‐out reaming is performed to the planned diameters. The sockets are then cleared of debris and refined. Tibial socket preparation is performed under arthroscopic visualization by identifying bony landmarks around the tibial footprint and marking the intended AMB and PLB socket apertures within the C‐shaped remnant region. Guided by a tibial aimer, 3.5 mm guide pins are advanced from the tibial cortex to create guide tunnels. Tibial sockets are then created from inside the joint using a retrograde reamer to match the graft diameter and planned socket length. Each graft is passed into its corresponding femoral socket and then into its respective tibial socket over guide wires. Femoral‐ and tibial‐side cortical buttons are seated on the lateral femoral cortex and the tibial cortex. Final fixation is completed with bundle‐specific tensioning, securing the PLB near extension and the AMB at greater flexion to reproduce the native reciprocal tension pattern. The knee is cycled through full range of motion to confirm appropriate graft tension and absence of intercondylar notch impingement before final tightening and securing of the adjustable loops. Video content can be viewed at https://doi.org/10.1002/atn2.70172.

Arthroscopic anterior cruciate ligament (ACL) reconstruction (ACLR) is considered the gold standard for treating ACL injuries and is most commonly performed using the single‐bundle (SB) technique and the double‐bundle (DB) technique. In recent years, the all‐inside technique (AIT) has gained widespread adoption for ACLR.^1^, ^2^ Unlike conventional full‐tunnel techniques, the AIT uses bone sockets with cortical suspensory fixation to preserve bone stock, reduce the required graft length, and facilitate preparation of a larger‐diameter graft, while maintaining reliable knee stability and reducing postoperative pain.^1^, ^2^

The DB technique creates separate full‐length tunnels to reconstruct the anteromedial bundle (AMB) and posterolateral bundle (PLB), which more closely replicates the native DB function of the ACL and provides superior rotational stability compared with the SB technique.^3^ In addition, it achieves clinical outcomes comparable to the SB technique and has been reported to provide lower revision rates and higher return‐to‐sport rates in athletes and other high‐demand patients.

However, the conventional 4‐tunnel DB technique presents distinct limitations. Creating 2 full‐length femoral and 2 full‐length tibial tunnels inevitably removes more bone and disrupts cortical integrity, and tunnel enlargement and coalescence have been frequently reported after such procedures.^4^, ^5^, ^6^ Furthermore, because the available autograft is split into 2 bundles and passed through 4 full‐length tunnels, each bundle is typically smaller in diameter than in AIT, which is associated with a higher risk of revision.^7^, ^8^ The bone‐sparing, socket‐based nature of AIT may help mitigate these limitations.^9^

In this technique note, we describe a modified all‐inside DB technique using 2 four‐strand hamstring tendon autografts, which integrates the advantages of the AIT with the biomechanical stability of the DB technique, incorporating some technical refinements. This procedure aims to better reproduce the native ACL anatomy and bundle‐specific tension patterns, reduce bone loss associated with full‐length tunnels, with the ultimate goal of decreasing residual anteroposterior and rotational instability and improving return‐to‐sport rates.

## SURGICAL TECHNIQUE

The complete technique is shown in Video 1. The pearls and pitfalls of this technique are shown in Table 1. The advantages and disadvantages of this modified procedure are summarized in Tables 2 and 3.

**TABLE 1:** The Key Surgical Steps of the Technique

Step	Pearls	Pitfalls
Graft preparation	Plan graft folding according to socket depth: short sockets allow more flexibility to fold hamstrings into 4‐strand bundles while maintaining adequate intra‐articular length. Pre‐mark the grafts for intraosseous segments.	Overfolding the graft without accounting for socket depth may lead to insufficient intra‐articular length or difficulty seating the graft fully. Underestimating required graft length can compromise fixation or tensioning.
Portal placement	Use a 30° arthroscope and a high AL viewing portal, plus an AMa portal, to visualize the entire femoral footprint and intercondylar notch.	Inadequate visualization from suboptimal portals can lead to nonanatomic femoral tunnel placement or posterior wall blowout. Do not rely solely on the standard AM portal for drilling both bundles.
Femoral footprint identification	Clearly mark the centers of the AMB and PLB footprints on the lateral wall using a probe or microfracture awl before drilling.	Misidentifying the footprint is a leading cause of ACLR failure. Avoid drilling based only on “clock face” without direct footprint visualization.
Femoral socket creation (inside‐out)	Drill 2 inside‐out short sockets with diverging trajectories. Use a depth gauge to confirm socket length and posterior wall integrity.	Excessively convergent trajectories can cause socket coalition or loss of bone bridge. Over‐drilling may breach the cortex or blow out the posterior wall—stop and re‐check frequently with a probe and depth gauge.
Tibial socket creation (retrograde)	Use a tibial guide to target the anatomic AMB and PLB tibial footprints and create 2 independent sockets. Protect the anterior horn of the lateral meniscus during retrograde drilling.	Misplaced tibial sockets may cause roof impingement or compromise the lateral meniscus root. Avoid placing sockets too close together, which can lead to bone bridge fracture or tunnel coalition.
Independent tensioning of AMB and PLB	Adjust tension separately on each adjustable loop to avoid over‐constraint.	Fixing both bundles at the same angle and tension may over‐constrain extension or flexion. Excessive tension on either bundle can increase graft forces and risk loss of motion.
Notch impingement check	After fixation, fully range the knee and inspect the notch to ensure no graft impingement on the roof or PCL. Slight notchplasty may be performed only if clearly necessary.	Neglecting a final impingement check can result in graft abrasion and early failure. Excessive notchplasty, however, may remove valuable anatomic landmarks and weaken the lateral wall.

AMa, anteromedial accessory; ACLR, anterior cruciate ligament reconstruction; AL, anterolateral; AMB, anteromedial bundle; PCL, posterior cruciate ligament; PLB, posterolateral bundle.

**TABLE 2:** Advantages and Disadvantages of Posteromedial PLB

Advantages
• Facilitates localization using reliable osseous landmarks, specifically by being close to the medial intercondylar eminence
• Reduces the risk of iatrogenic injury to the anterior horn of the lateral meniscus
• Optimally aligns with the C‐shaped ACL tibial footprint
• The medialized tibial tunnel may provide biomechanical advantages
• The medialized tibial tunnel may be associated with better clinical stability

Disadvantages
• Risk of injuring the medial intercondylar ridge due to inaccurate positioning
• Potential for injury to the surrounding soft tissues on the posteromedial side

ACL, anterior cruciate ligament; PLB, posterolateral bundle.

**TABLE 3:** Advantages and Limitations of the Modified All‐Inside DB Technique

Advantages
• Open incision facilitates tendon harvesting, provides greater working space, and allows for more precise tunnel positioning.
• The socket technique maximizes bone stock preservation.
• The DB four‐strand structure provides superior rotational stability, leading to a higher rate of successful RTS.
• Adjustable‐loop titanium cortical button fixation ensures optimal graft‐bone contact and facilitates precise tension adjustment.
• Anatomical PLB positioning is more compliant with the native ACL tibial footprint structure.

Limitations
• Open incision carries potential for increased infection risk, greater postoperative pain and results in an inferior cosmetic outcome compared with the AIT.
• DB tunnels carry a risk of tunnel convergence.
• This technique has a higher cost and requires a high level of surgical expertise.

ACL, anterior cruciate ligament; AIT, all‐inside technique; DB, double‐bundle; PLB, posterolateral bundle; RTS, return to sport.

### Surgical Indications

This technique is applicable to patients meeting the following criteria:^10^ (1) ACL tibial footprint anteroposterior diameter > 14 mm, (2) intercondylar notch width > 12 mm, (3) no severe bone loss or bone contusion, (4) Kellgren–Lawrence grade ≤ III osteoarthritis, and (5) no concomitant ligament injury of the knee.

### Preparation of the AMB and PLB Grafts

**FIGURE 1:**
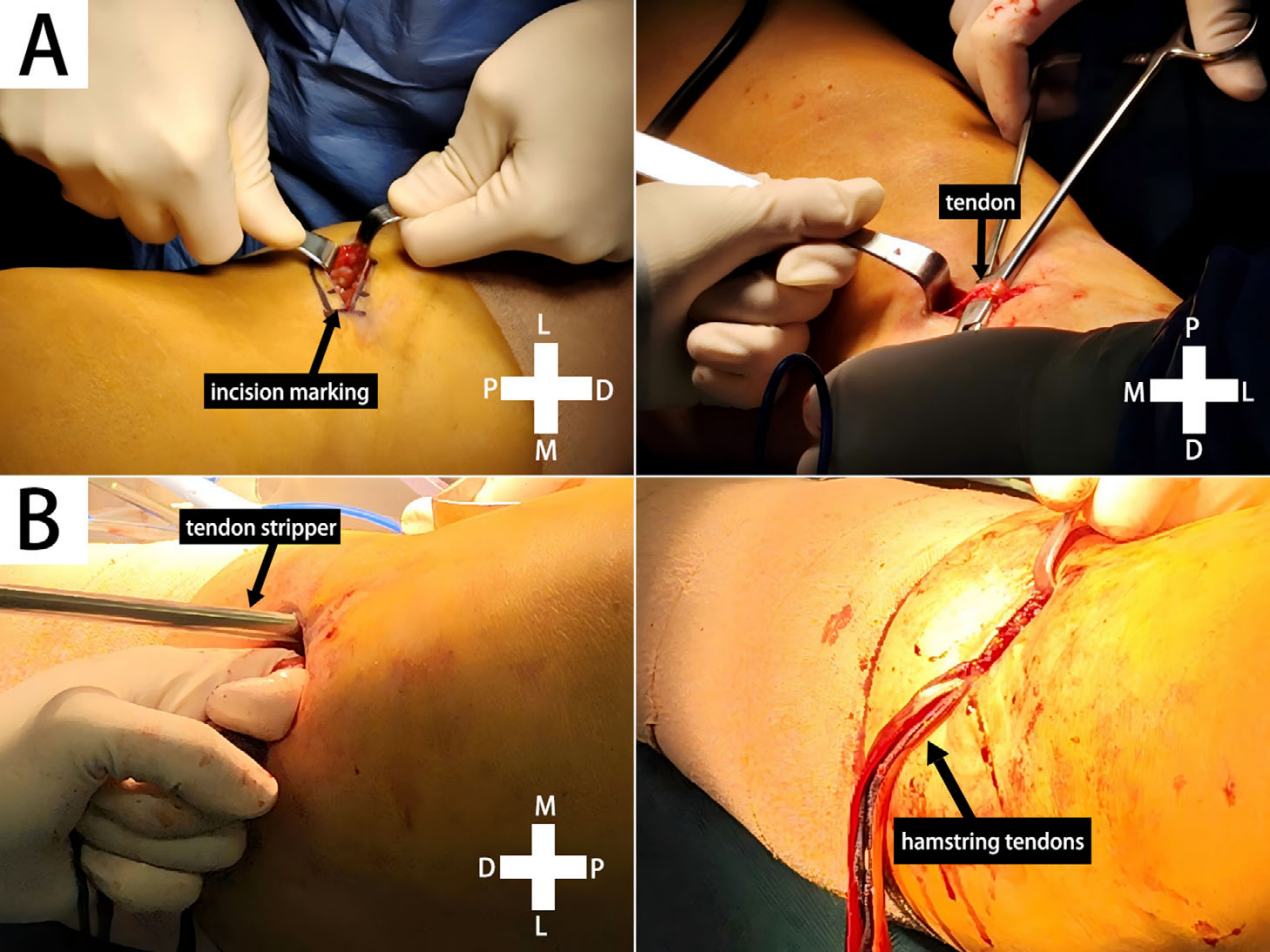
Tendon harvest procedure (left knee illustrated). (A) A small oblique incision, approximately 3 cm in length, is made 2 cm medial to the tibial tubercle and 4 cm distal to the joint line. Preoperative markings indicate the incision line, with short perpendicular lines used for skin alignment during closure. Tissues are dissected layer by layer to identify and separate the hamstring tendons from surrounding attachments. (B) A tendon stripper is then used to detach the hamstring tendons from their proximal attachments. Orientations are labeled as P (proximal), D (distal), L (lateral), and M (medial).

**FIGURE 2:**
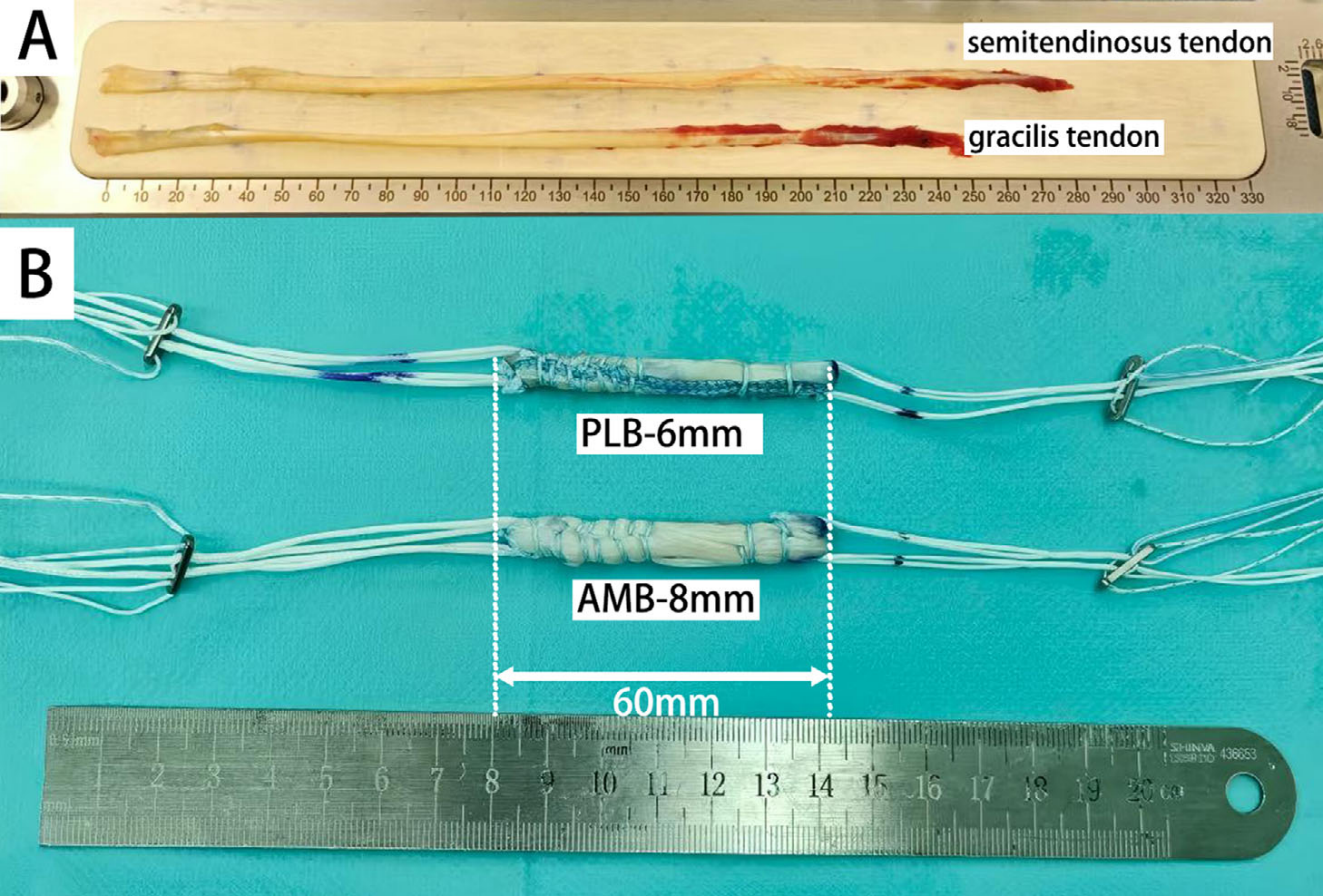
Graft preparation. (A) The harvested hamstring tendons are shown. (B) Using the GraftLink technique, the tendons are braided into double‐bundle four‐strand grafts. The resultant 2 bundles are each approximately 60 mm in length, with the AMB measuring approximately 8 mm in diameter and the PLB measuring approximately 6 mm in diameter. Both bundles are connected to adjustable‐loop titanium cortical buttons on both the proximal and distal ends. (AMB, anteromedial bundle; PLB, posterolateral bundle.)

A 3 cm oblique incision is made approximately 2 cm medial to the tibial tuberosity and 4 cm distal to the joint line. The hamstring tendons are harvested with a tendon stripper (Figure 1). The tendons are prepared and fashioned into the AMB and PLB using the GraftLink technique. Both grafts measure approximately 60 mm in length, and their ends are secured to adjustable‐loop titanium cortical buttons (Arthrex, Naples, FL) (Figure 2).

### Arthroscopy Examination

**FIGURE 3:**
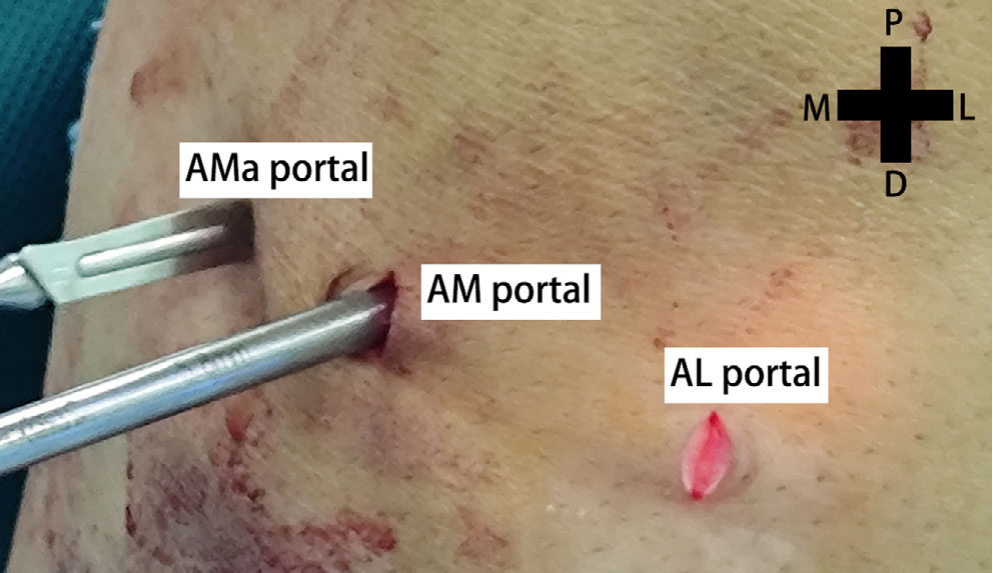
Arthroscopic portal placement (left knee illustrated). The AMa portal, AM portal, and AL portal are established. Orientations are labeled as P (proximal), D (distal), L (lateral), and M (medial). (AMa, anteromedial accessory; AL, anterolateral; AM, anteromedial.)

With the knee flexed at 90°, 3 portals are created: an anteromedial accessory portal, an anteromedial portal, and an anterolateral portal (Figure 3). Arthroscopy with a 30° arthroscope (Arthrex, Naples, FL) is performed to confirm the ACL tear. The remnant fibers are debrided.

### Femoral Socket Creation

**FIGURE 4:**
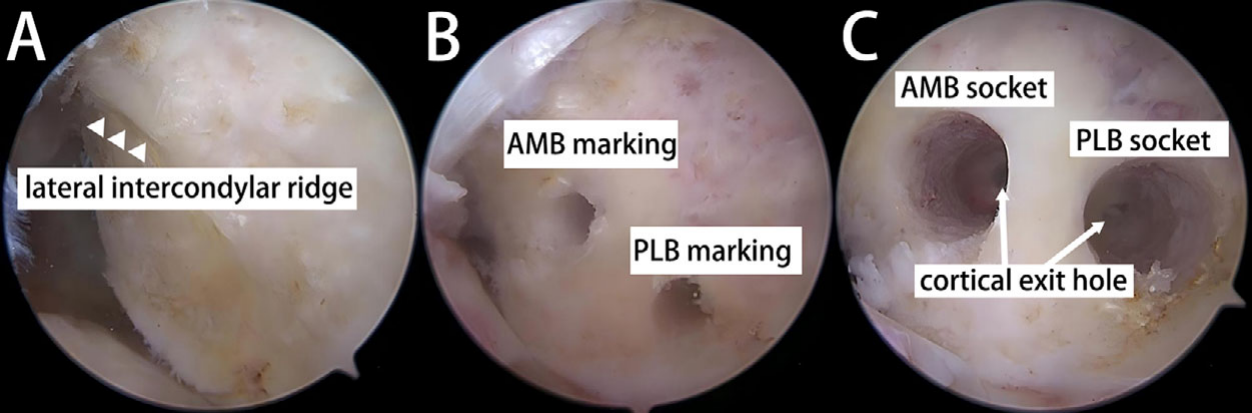
Femoral tunnel creation, showing the medial wall of the lateral femoral condyle of the left knee. (A) Identifying the bony landmark of the native ACL femoral footprint, specifically the lateral intercondylar ridge. (B) Tunnel positioning is performed using a microfracture awl while the knee is flexed at 90° and allowed to hang naturally. The AMB socket aperture is positioned at the junction of the posterior femoral condyle cartilage and the intercondylar ridge. The PLB socket aperture is located at the intersection of the lateral intercondylar ridge and the coronal plane defined by the anterior edge of the posterior horn of the lateral meniscus. (C) With the knee in the maximum hyperflexed position, the AMB and PLB sockets are drilled anterogradely using a drill bit matching the graft diameter. The socket length is determined according to the length of the prepared graft. The small guide‐wire aperture leading to the lateral femoral cortex is visible within the socket. (AMB, anteromedial bundle; PLB, posterolateral bundle.)

The femoral sockets are established first. With the knee flexed at 90°, the PLB footprint is identified and marked at the junction of the intercondylar ridge and the coronal plane through the anterior margin of the posterior horn of the lateral meniscus. The AMB footprint is identified at the junction of the posterior cartilage margin of the femoral condyle and the intercondylar ridge (Figure 4A). Bone markings are created at the footprint centers using a microfracture awl (Wuyang Medical Instrument, Hefei, Anhui) (Figure 4B).

With the knee placed in hyperflexion, a guide pin is advanced anterogradely. Under direct visualization, both the PLB and AMB guide tunnels are drilled, and their lengths are measured. Based on the graft diameter and length, sequential reaming is performed over the guide pin with progressively larger cannulated reamers until the desired graft diameter is achieved, progressing from inside to outside. The bone sockets are gradually enlarged to the planned diameter and depth (Figure 4C).

### Tibial Socket Creation

**FIGURE 5:**
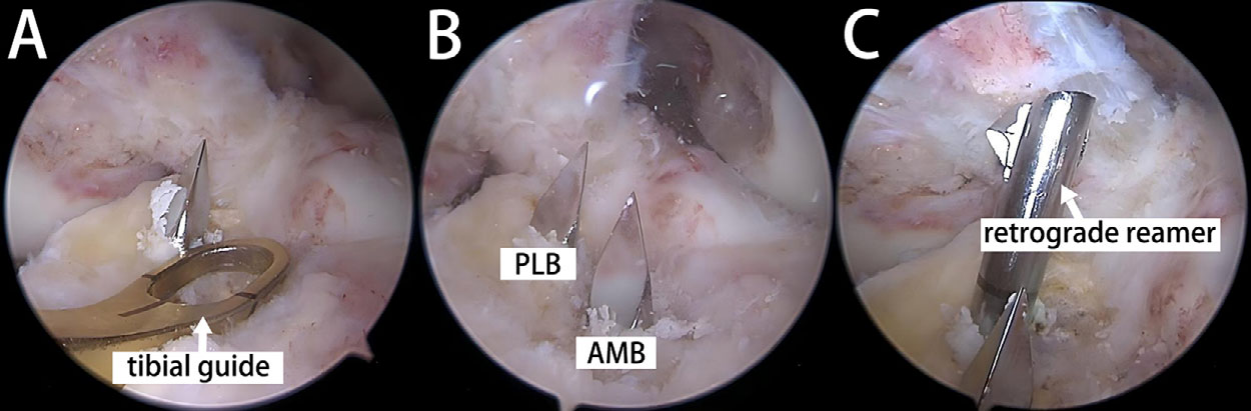
Tibial tunnel creation, showing the tibial plateau of the left knee joint. (A) The tibial aimer is used to individually locate the PLB and AMB positions. (B) Small‐diameter full‐length tunnels are drilled anterogradely. (C) Bone sockets are drilled retrogradely using a retrograde reamer that matches the graft diameter. The socket length is determined according to the length of the prepared graft. (AMB, anteromedial bundle; PLB, posterolateral bundle.)

The anterior and medial bony landmarks of the tibial footprint are identified, and the tunnel positions are marked within the C‐shaped region. Using a tibial aimer (Rejoin Mastin Medical Instrument, Hangzhou, Zhejiang), guide pins are advanced to create the AMB and PLB tunnels (Figure 5A,B). Retrograde reaming (Arthrex, Naples, FL) is then performed to form bone sockets corresponding to the graft diameter and length (Figure 5C).

### Graft Passage and Fixation

The grafts, equipped with tail suture loops and adjustable‐loop titanium cortical buttons, are passed through the femoral tunnels. After tensioning the grafts, the adjustable‐loop titanium cortical buttons are fixed on the lateral femoral cortex. The same technique is used to pass the grafts with the adjustable‐loop titanium cortical buttons through the tibial tunnels under guide‐wire assistance.

The knee is repeatedly flexed and extended to ensure the grafts are properly tensioned. The PLB is tensioned and fixed at full extension, whereas the AMB is tensioned and fixed at approximately 60° of knee flexion. After confirming graft tension and the absence of notch impingement, all adjustable‐loop titanium cortical buttons are then tightened and secured. Arthroscopic visualization confirms there is no impingement between the grafts and the femoral intercondylar notch.

### Ethics Approval

All procedures performed in this study involving human subjects were in accordance with the ethical standards of the institutional and/or national research committee and with the 1964 Helsinki Declaration and its later amendments or comparable ethical standards. This study has been approved by the ethic committee of the Eighth Affiliated Hospital of Sun Yat‐sen University. Informed consent was obtained from the subjects.

## DISCUSSION

We describe a modified anatomical all‐inside DB ACLR technique that integrates the advantages of the AIT and DB technique. The technique is feasible and reproducible in a clinical setting.

The AIT offers notable advantages due to its minimally invasive nature, including maximized bone stock preservation, reduced postoperative pain, and improved cosmetic outcomes.^1^ The all‐inside DB technique was first reported in 2008.^9^ However, the technical complexity of this approach and its higher cost may have limited its subsequent development and wider adoption. Nevertheless, as a technique that combines the advantages of the AIT and DB technique, this concept remains clinically relevant.

Our modified technique effectively integrates the advantages of both the AIT and conventional DB technique. The use of bone sockets facilitates bone stock preservation, and using adjustable‐loop titanium cortical buttons allows for precise graft tensioning. Biomechanical studies have consistently showed that the DB technique is superior to the SB technique in restoring knee kinematics, particularly in enhancing rotational stability.^11^ Several recent high‐quality clinical studies have reported that patients undergoing DB technique showed better anterior and rotational knee stability, lower graft failure rates, and higher return‐to‐sport rates than those treated with the SB technique. In addition, we incorporated several pragmatic refinements to the surgical technique.

To simplify the procedure, we elected to retain a traditional small open hamstring harvest. This approach provides clear anatomical landmarks and facilitates reliable graft harvesting. It also allows wide exposure of the pes anserinus region and the anteromedial tibial cortex, which facilitates guide placement and angle adjustment during tibial socket creation. This exposure enables more precise intraoperative control of bone‐bridge width and tunnel trajectories and may help reduce the risk of tunnel coalition and other technical complications.^12^ Performing these key steps under direct visualization may also improve efficiency and help avoid excessive operative time.

For femoral tunnel preparation, we did not adopt a retrograde outside‐in drilling technique, which is normally used in AIT. Given the critical importance of femoral tunnel position for ACLR,^13^ and considering that inter‐tunnel bone‐bridge width and fixation strategy can substantially influence the structural stability of the graft–femur complex in DB technique,^10^ we preferred to create both femoral sockets from inside the joint under direct visualization. An inside‐out approach may facilitate more accurate anatomic socket placement and control of tunnel separation and bone‐bridge width in DB technique, potentially reducing the risk of bone‐bridge fracture and tunnel coalition.

**FIGURE 6:**
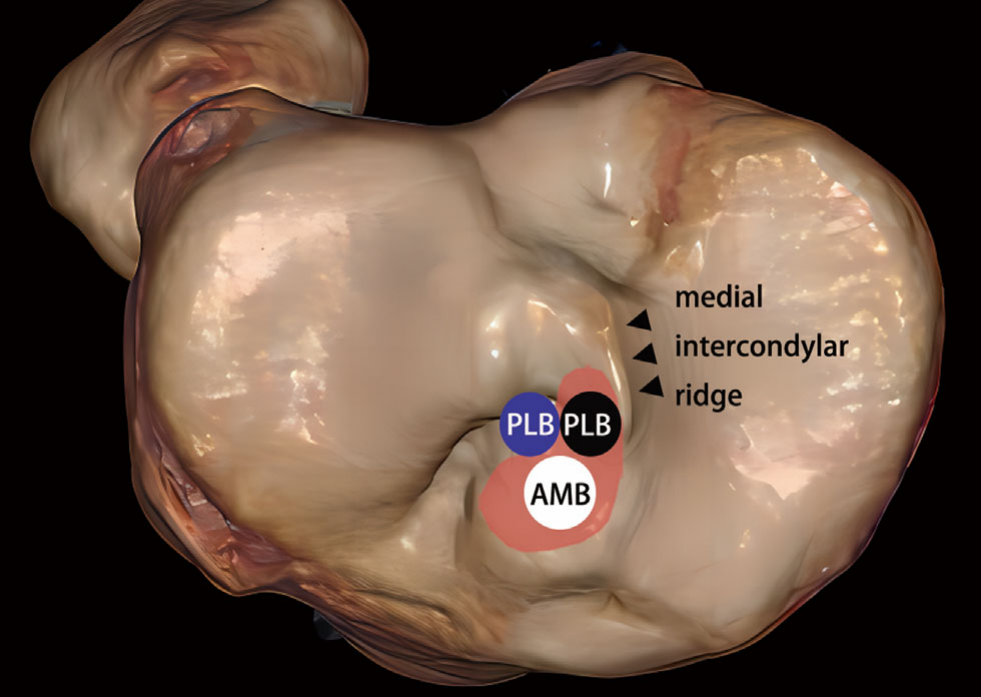
Anatomical PLB placement in the tibial C‐shaped region. A superior view of the tibial plateau of the right knee joint is shown. The red region indicates the C‐shaped structure at the ACL tibial footprint. In traditional reconstruction, the conventional PLB (purple region) is positioned posterolaterally to the AMB (white region). In our modified DB technique, the PLB (black region) is placed posteromedial side of the AMB, closer to the medial intercondylar eminence, and both bundles are located within the C‐shaped region. (AMB, anteromedial bundle; PLB, posterolateral bundle.)

For tibial socket placement, the PLB socket is positioned slightly posteromedial to the AMB socket so that both bundles are located within the C‐shaped direct‐fiber portion of the ACL tibial footprint (Figure 6). Anatomic and histologic studies have suggested that a posteromedial placement of the PLB within the tibial footprint may better reproduce its native structure.^14^, ^15^ Biomechanical evidence suggests that excessive laterally misplaced PLB may lead to increased graft laxity.^16^

During graft fixation, the AMB and PLB are tensioned and fixed at different knee flexion angles, with the PLB secured near full extension and the AMB fixed at greater flexion. This bundle‐specific tensioning strategy is intended to reproduce the native reciprocal tension pattern of the 2 ACL bundles across the range of motion, thereby facilitating restoration of physiological knee biomechanics while minimizing the risks of residual laxity and overconstraint.

In conclusion, this technique note describes a modified anatomical all‐inside DB ACLR technique that integrates the advantages of AIT and DB technique while incorporating pragmatic refinements in graft harvesting, tunnel placement, and bundle‐specific tensioning.

## DECLARATION OF GENERATIVE AI AND AI‐ASSISTED TECHNOLOGIES IN THE WRITING PROCESS

During the preparation of this work, the authors used ChatGPT (OpenAI) in order to translate parts of the manuscript and improve English language clarity and style. The authors reviewed, edited, and verified all content generated by the tool and take full responsibility for the content of the publication.

## DISCLOSURES

The authors (H.Y., H.L., Y.C., W.Y., W.Z., M.M., Y.J.) declare the following financial interests/personal relationships which may be considered as potential competing interests: H.Y. reports that financial support was provided by the Guangdong Provincial Clinical Research Center for Orthopedic Diseases; National Natural Science Foundation of China; Natural Science Foundation of Guangdong Province; Shenzhen Science and Technology Program; Futian Healthcare Research Project; and The Eighth Affiliated Hospital Clinical Research Program, Sun Yat‐Sen University. H.L. reports that financial support was provided by the Guangdong Provincial Clinical Research Center for Orthopedic Diseases; National Natural Science Foundation of China; Natural Science Foundation of Guangdong Province; Shenzhen Science and Technology Program; Futian Healthcare Research Project; and The Eighth Affiliated Hospital Clinical Research Program, Sun Yat‐Sen University. Y.C. reports that financial support was provided by the Guangdong Provincial Clinical Research Center for Orthopedic Diseases; National Natural Science Foundation of China; Natural Science Foundation of Guangdong Province; Shenzhen Science and Technology Program; Futian Healthcare Research Project; and The Eighth Affiliated Hospital Clinical Research Program, Sun Yat‐Sen University. W.Y. reports that financial support was provided by the Guangdong Provincial Clinical Research Center for Orthopedic Diseases; National Natural Science Foundation of China; Natural Science Foundation of Guangdong Province; Shenzhen Science and Technology Program; and Futian Healthcare Research Project; and The Eighth Affiliated Hospital Clinical Research Program, Sun Yat‐Sen University. W.Z. reports that financial support was provided by the Guangdong Provincial Clinical Research Center for Orthopedic Diseases; National Natural Science Foundation of China; Natural Science Foundation of Guangdong Province; Shenzhen Science and Technology Program; Futian Healthcare Research Project; and The Eighth Affiliated Hospital Clinical Research Program, Sun Yat‐Sen University. M.M. reports that financial support was provided by the Guangdong Provincial Clinical Research Center for Orthopedic Diseases; National Natural Science Foundation of China; Natural Science Foundation of Guangdong Province; Shenzhen Science and Technology Program; Futian Healthcare Research Project; and The Eighth Affiliated Hospital Clinical Research Program, Sun Yat‐Sen University. Y.J. reports that financial support was provided by the Guangdong Provincial Clinical Research Center for Orthopedic Diseases; National Natural Science Foundation of China; Natural Science Foundation of Guangdong Province; Shenzhen Science and Technology Program; Futian Healthcare Research Project; and The Eighth Affiliated Hospital Clinical Research Program, Sun Yat‐Sen University.

## FUNDING

This work was supported by the Guangdong Provincial Clinical Research Center for Orthopedic Diseases (2023B110001), National Natural Science Foundation of China (82203740), Natural Science Foundation of Guangdong Province (2023A1515010552), Shenzhen Science and Technology Program (JCYJ20230807111307015), Shenzhen Science and Technology Program (KCXFZ20230731093001002), Futian Healthcare Research Project (FTW2025023), and The Eighth Affiliated Hospital Clinical Research Program, Sun Yat‐Sen University (ZDBY‐IIT‐202403‐54).
